# Cervical necrosis after chemoradiation for cervical cancer: case series and literature review

**DOI:** 10.1186/1748-717X-8-220

**Published:** 2013-09-23

**Authors:** Ziad Simon Fawaz, Maroie Barkati, Marie-Claude Beauchemin, Philippe Sauthier, Philippe Gauthier, Thu Van Nguyen

**Affiliations:** 1 Department of Radiation Oncology and Gynecologic Oncology, Notre-Dame Hospital, University of Montreal Hospital Center (CHUM), Montreal, Canada

**Keywords:** Cervical cancer, Necrosis, Radiotherapy, Radiation therapy, Chemoradiation, Smoking, Management

## Abstract

**Background:**

The aim of this study was to assess the management of cervical necrosis (CN) following radiotherapy (RT) and the impact of smoking status. This rare complication mimics a neoplastic recurrence, and causes concern among attending physicians.

**Methods:**

Between July 2008 and March 2013, 5 women on 285 with localized cervical cancer had a CN following RT. Patients were treated with concomitant chemoradiation. The medical records were reviewed to abstract demographic and clinical information until March 2013.

**Results:**

1.75% (95% confidence interval: 0.23 to 3.28%) developed CN. All patients were smokers with a mean of 19.5 pack-years (range: 7.5-45 pack-years). All patients were treated with weekly Cisplatin chemotherapy and external beam radiation to the pelvis, 45 Gy in 25 fractions. Four patients received an extra boost with a median dose of 7.2 Gy (range: 5.4-10 Gy). All patients had intracavitary brachytherapy (range: 27.9 to 30 Gy). Clinical presentation was similar for all the cases: vaginal discharge associated with pain. Mean time for time post-radiation therapy to necrosis was 9.3 months (range: 2.2-20.5 months). Standard workup was done to exclude cancer recurrence: biopsies and radiologic imaging. Conservative treatment was performed with excellent results. Resolution of the necrosis was complete after a few months (range: 1 to 4 months). Median follow-up until March 2013 was 19 months. All the patients were alive with no clinical evidence of disease.

**Conclusions:**

This study, the largest to date, shows that conservative management of CN after RT is effective, and should be attempted. This complication is more common in smokers, and counseling intervention should result in fewer complications of CN.

## Background

Chemoradiation, where platinum-based chemotherapy is given concomitantly with radiotherapy, is considered to be the standard of care for the treatment of locally advanced cervical cancer since the clinical announcement of the National Cancer Institute (NCI) in 1999. Mild and acute side effects are experienced by most patients, and are generally transitory [1-3]. Severe long-term toxicity, accounting for 1% to 3% [4], is well documented [5,6], and involves gastrointestinal and urological systems: distal ureteral necrosis, distal ureteral stenosis, vesico-vaginal fistula, vesico-intestinal fistula, severe fibrotic bladder shrinkage, urethral stenosis, and bladder necrosis [7,8].

*Cervical necrosis* (CN) associated with *radiation therapy* (RT) for cervical cancer has also been described. Since 1986, 5 case reports, the largest of which consisted of 3 patients, have been published in further support of CN following RT for a total of only 12 cases described in the literature [9-13]. Interest in this field comes from more than half a century ago with a case report on uterine necrosis following irradiation for cervical carcinoma [14]. However, radiation therapy treatment has changed since then, and limited information in the oncologic literature is available for the clinicians on the management of this complication, with most of the studies suggesting laparoscopic examination and surgical intervention.

In this study, the largest series to date of patients who experienced that complication, the late toxic reaction of cervical necrosis is described with an emphasis on the clinical course and conservative management of this toxicity. This rare complication mimics a neoplastic recurrence, and causes concern among attending physicians.

## Methods

Between July 2008 and March 2013, five women had a cervical necrosis following radiation therapy for a localized cervical cancer, which includes confined cancers with stages up to IIIB. During this period, 285 patients with localized cervical cancer were treated with concomitant chemoradiotherapy at the Department of Radiation Oncology of the Notre-Dame Hospital of the University of Montreal Hospital Center (Montreal, Canada). The medical records of the patients were then reviewed, and analyzed to abstract demographic and clinical information through the oncological data archiving system at our center.

Following a careful and thorough history taking and physical examination with investigation and biopsy, the diagnosis for cervical cancer was made. Patients were treated with concurrent chemoradiation with weekly Cisplatin at a dose of 40 mg/m^2^ for radiosensitisation. Our standard protocol in our centre consisted of external beam radiotherapy (EBRT) to the whole pelvis. Patients with involved para-aortic nodes on imaging were treated using an extended field technique. For pelvic or para-aortic nodal involvement, a boost was added. Intracavitary brachytherapy consisted of high-dose rate (HDR) brachytherapy and was usually started during EBRT. The procedure was performed by one of three trained radiation oncologists at our center, and by a radiation oncologist from another center for one patient. A prescription was then given to the patients: vaginal topical emollients for moisturizing, twice-weekly estradiol vaginal tablets, and using a vaginal dilator every day, except when there is sexual intercourse, to prevent and reverse the vaginal shrinkage. Patients were then followed alternately by the gynecologic oncologist and the radiation oncologist every 3 to 4 months in the first 2 to 3 years, and then every 6 months up to 5 years, and thereafter annually indefinitely. A follow-up positron emission tomography (PET) scan was done 4 to 6 months after the radiotherapy.

At presentation with clinical features of the necrosis, the clinical presentation was noted, as well as the date of diagnosis for the necrosis. The time post-radiation therapy before the necrosis was calculated in months. A cervical biopsy was done for each patient, as well as imaging by magnetic resonance imaging (MRI) or PET scan to rule out a recurrence of the cancer. Management and treatment of this complication were done, and patients were followed-up thereafter. Post-treatment clinical improvement, smoking cessation after counseling and post-coital bleeding were also evaluated. The last follow-up for all 5 patients was in March 2013.

In brief, the following parameters were analyzed: age at diagnosis, stage, initial tumor size in centimeters, history of abdominal surgery, smoking status, chemotherapy received, radiation therapy delivered to the pelvis, boost to lymph nodes, brachytherapy, date of last treatment, clinical presentation, date of diagnosis for the necrosis, time post-radiotherapy before necrosis in months, cervical biopsy with the results, imaging with MRI or PET scan, treatment of the necrosis, time to clinical improvement of the necrosis after the treatment in months, smoking cessation or not after counseling, post-coital bleeding after the necrosis, time of follow-up in months, and status in March 2013.

## Results

The characteristics for the parameters of the 5 cases included in the study are shown in Table 1. Of the 285 women treated during this period, 5 (1.75%, 95% confidence interval: 0.23 to 3.28%) developed CN after chemoradiation for cervical carcinoma. Mean age at diagnosis of the CN was 36.4 years old (range: 28–49), while mean age among the 285 patients was 51.45 years old (95% confidence interval: 49.97 to 52.92 years old). The patients had cancer stages ranging from IB-2 to IIIB with a mean initial tumor size of 5.3 cm (range: 2.8-8.5), compared to 5.36 cm (95% confidence interval: 5.11 to 5.60 cm) for all the patients treated during that period. None had a history of abdominal surgery. Active smoking was found in 181 of the 285 patients, representing 63.51% (95% confidence interval: 57.92 to 69.10%). Of the 181 smokers, five have developed the CN, representing 2.76% (95% confidence interval: 0.37 to 5.15%). All patients with CN were smokers with a mean of 19.5 pack-years (range: 7.5-45). All patients were treated with weekly Cisplatin chemotherapy (4 received 6 cycles; 1 received 7 cycles). The dose for external beam radiation to the pelvis was the same for the 5 patients, 45 Gy in 25 fractions. 4 patients received an extra boost of radiation to involved lymph nodes with a median dose of 7.2 Gy (range: 5.4-10 Gy), and 1 patient did not get an extra boost because she had no lymph node involvement. All patients had intracavitary brachytherapy for doses ranging from 27.9 to 30 Gy in 4 or 5 treatments. Table 2 shows the brachytherapy dosimetry details with the surface doses to the colpostats.

**Table 1:** Characteristics of patients with cervical necrosis following radiation therapy for cervical cancer

**Characteristics**	**1**	**2**	**3**	**4**	**5**
**Age at diagnosis**	28	36	35	49	34
**Stage**	IB-2	IB-2	IIB	IIB	IIIB
**Initial tumor size (cm)**	8.5	5.6	3.9	2.8	5.6
**History of surgery**	No	No	No	No	No
**Smoking**	Yes (7,5 p-y)	Yes (45 p-y)	Yes (10 p-y)	Yes (20 p-y)	Yes (15 p-y)
**Chemotherapy**	Cisplatin ×6	Cisplatin ×6	Cisplatin ×6	Cisplatin ×7	Cisplatin ×6
**RT (pelvis)**	45 Gy in 25 fx	45 Gy in 25 fx	45 Gy in 25 fx	45 Gy in 25 fx	45 Gy in 25 fx
**Boost lymph nodes**	5,4 Gy in 3 fx (pelvic)	5,4 Gy in 3 fx (pelvic)	0 Gy (No LN)	10 Gy (pelvic)	9 Gy in 5 fx (pelvic)
**Brachytherapy**	30 Gy in 5 tx	28 Gy in 4 tx	30 Gy in 5 tx	30 Gy in 5 tx	27,9 Gy in 4 tx
**Date of last treatment**	2008-07-23	2011-08-25	2010-12-23	2011-12-15	2012-04-16

*RT* Radiation therapy, *Gy* Gray, *fx* Fractions, *tx* Treatments, *LN* Lymph node, *p-y* Pack-years.

**Table 2:** Brachytherapy dosimetry details: surface doses to the colpostats in Gray

**Fractions**	**1**	**2**	**3**	**4**	**5**
**Fx 1**	R : 6.34	R : 8.64	R : 6.70	R : 9.01	R : 7.6
	L: 6.36	L : 8.11	L : 6.82	L : 9.09	L : 12.9
**Fx 2**	R : 6.58	R : 8.29	R : 6.74	R : 8.39	R : 9.1
	L : 6.57	L : 8.46	L : 6.74	L : 9.12	L : 11.7
**Fx 3**	R : 6.60	R : 8.41	R : 6.61	R : 10.79	R : 8.6
	L : 6.63	L : 8.24	L : 6.61	L : 10.80	L : 9.7
**Fx 4**	R : 6.51	R : 8.36	R : 6.98	R : 9.79	R : 8.8
	L : 6.58	L : 8.21	L : 7.06	L : 10.81	L : 9.8
**Fx 5**	R : 6.61		R : 6.65	R : 10.98	
	L : 6.52		L : 6.71	L : 10.92	

*Fx* Fraction, *R* Right, *L* Left.

**Figure 1:**
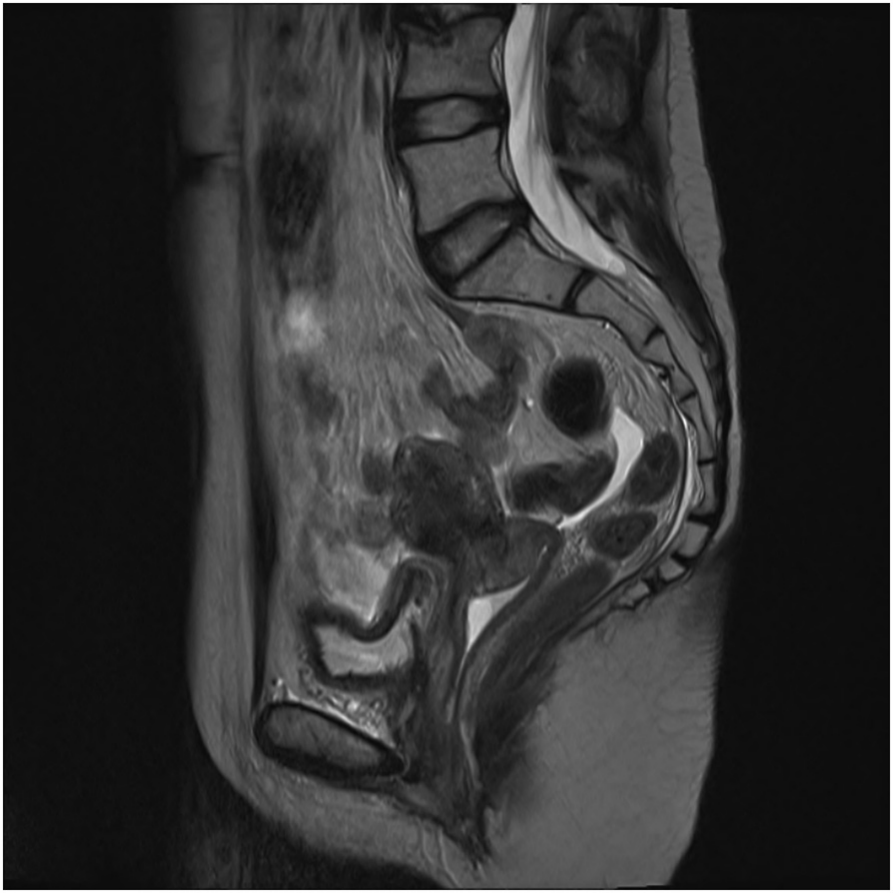
Patient #1: MRI at time of cervical necrosis (January 2009).

**Figure 2:**
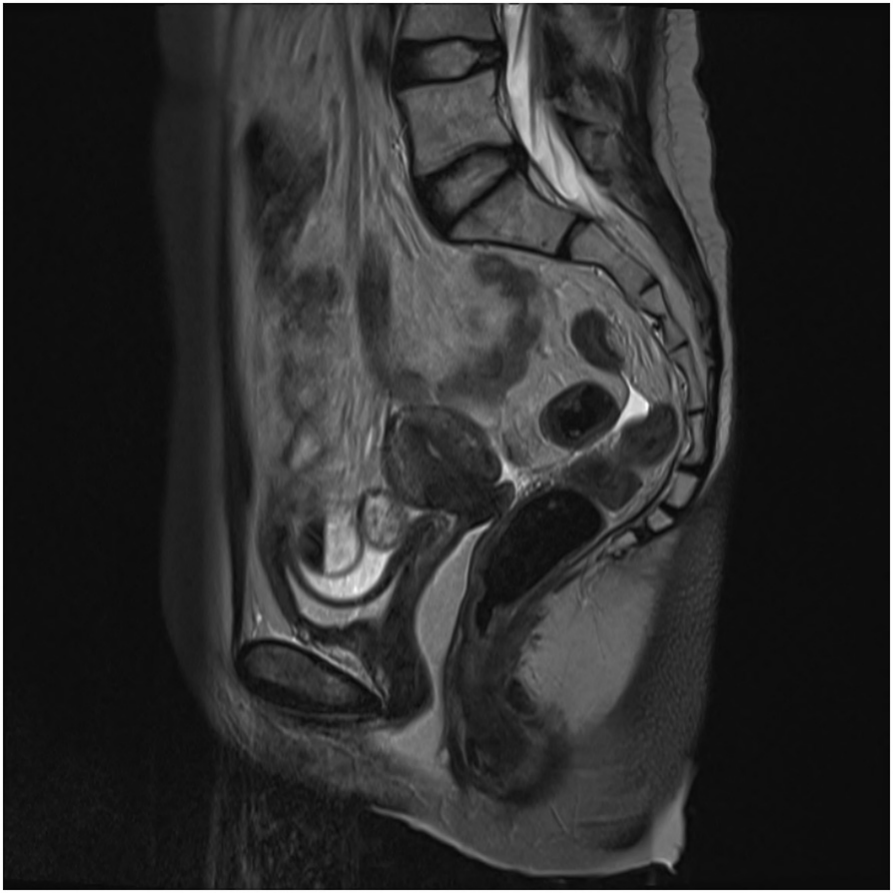
Patient #1: MRI 3 months after treatment of cervical necrosis (April 2009).

Clinical presentation was similar for all the patients: vaginal discharge associated with pelvic or abdominal pain for at least 1 week before consultation. Vaginal bleeding was also seen in two patients. Time post-radiation therapy to necrosis was calculated, and the mean time was 9.3 months (range: 2.2-20.5). Cervical biopsy was done for every patient. The results were similar, and correlate with post-radiotherapy changes: necroinflammatory exudate with the absence of neoplasia was found in the 5 patients at the cervical biopsy. Imaging was done, either a MRI or a PET scan, to assess whether it was a recurrence or not. 2 patients had a PET scan exclusively, and 3 had a MRI and a PET scan. MRI images for patient #1 at time of cervical necrosis, and 3 months after, are shown in Figures 1 and 2. Treatment consisted of: counseling for smoking cessation for all patients, and vaginal douching. Metronidazole was used in 3 patients. Opioid analgesics, hydromorphone or morphine, were used to control the pain for 3 patients. One patient has also received pregabalin and amitriptyline for pain management.

All patients were assessed one month after the diagnosis of CN, and then, every 2 to 6 months. Median follow-up until March 2013 was 19 months with a range from 11 to 56 months, as shown in Table 3. Clinical improvement of the necrosis after the treatment was assessed, and was positive in 4 patients (80%) at 1 month, and in 1 patient at 4 months (20%). All patients had complete resolution of the necrosis at 4 months. None had stopped smoking permanently after the smoking cessation counseling, but 1 patient had stopped temporarily for 3 months. Post-coital bleeding was evaluated in each patient, and only one patient did not experience this side effect. The bleeding lasted up to 1 month in 1 patient, up to 4 months in 1 patient, up to 1 year in 1 patient, and up to 2 years in the last patient. Finally, disease status was assessed in March 2013, and all the 5 patients were still alive with no clinical evidence of disease.

**Table 3:** Diagnosis, management and follow-up of the cervical necrosis

**Characteristics**	**1**	**2**	**3**	**4**	**5**
**Clinical presentation, necrosis**	Abdominal pain, vaginal discharge and minimal bleeding ×1 mo	Low back pain with vaginal discharge, no bleeding ×3 wk	Pelvic pain with significant vaginal discharge ×3 wk	Pelvic pain with foul vaginal discharge ×4 mo	Vaginal discharge with brown clots, stench ×1 wk
**Date of necrosis dx**	2009-01-09	2011-11-01	2012-09-06	2012-09-13	2013-01-22
**Time post-RT before necrosis (months)**	5.6	2.2	20.5	9.0	9.2
**Cervical biopsy**	Yes	Yes	Yes	Yes	Yes
**Cervical biopsy (results)**	Necroinflammatory exudate with reactive atypia	Necroinflammatory exudate with reactive atypia	Necroinflammatory exudate	Necroinflammatory exudate, post-radiotherapy changes	Necroinflammatory exudate with reactive atypia
**Imaging (MRI or PET scan)**	Yes (MRI, PET)	Yes (MRI, PET)	Yes (MRI, PET)	Yes (PET)	Yes (PET)
**Treatment**	Counseling for smoking cessation; Metronidazole	Counseling for smoking cessation; Morphine PRN; Pregabalin; Amitriptyline	Counseling for smoking cessation; Metronidazole; Morphine PRN	Counseling for smoking cessation; Vaginal douching; Morphine PRN	Counseling for smoking cessation; Metronidazole; Vaginal douching; Hydromorphone PRN
**Time to clinical improvement of the necrosis after treatment (months)**	1	1	4	1	1
**Smoking cessation after counseling**	No	No	No	Temporary (×3 mo)	No
**Post-coital bleeding**	Yes (FU 2 y)	Yes (FU 1 y)	Yes (FU 4 mo)	No	Yes (FU 1 mo)
**Time of follow-up (months)**	56	19	27	15	11
**Status in March 2013**	Alive, NED	Alive, NED	Alive, NED	Alive, NED	Alive, NED

*wk* Weeks, *mo* Months, *dx* Diagnosis, *MRI* Magnetic resonance imaging, *PET* Positron emission tomography, *FU* Follow-up, *N/A* Not applicable, *y* Year, *NED* No evidence of disease.

## Discussion

Cervical necrosis after chemoradiation is a significant complication. It reaches 1.75% of all women treated, and 2.76% of smokers who receive the same treatment modalities. Current knowledge of CN following RT for cervical cancer is based on 5 case reports with a total of 12 patients. Our case series, the largest to date, evaluates mainly the management of radiation necrosis and the smoking status impact on this complication.

Our data support the previously proposed theory that smoking promotes this kind of complication, since it limits the delivery of blood and oxygen to the body tissues [15-17]. In our study, 100% of patients with CN were smokers, while 63.51% of the whole cohort were active smokers. This difference of more than 36% reinforces the idea that smoking is related to the risk of necrosis. There might also be a correlation between the number of pack-years of smoking and the time post-radiation therapy before necrosis. In fact, the number of pack-years of smoking could be a surrogate for long-term poor tissue oxygenation, which, in turn, promotes the necrosis of tissues. However, the majority of tobacco smokers do not develop this complication, and our type of study does not allow us to draw such conclusions, but it would be interesting to assess this question in further studies to identify potential predisposing factors.

Therapies used in this study include counseling for smoking cessation, antibiotics such as metronidazole, hydrogen peroxide vaginal douches, and use of opioids such as hydromorphone and morphine. Smoking cessation and counseling intervention, a workup that was added to the NCCN Guidelines for cervical cancer in 2013, should be strengthened. All the patients were followed-up one month after the complication, and 4 out of 5 had a clinical improvement at the visit, and the last patient had a complete remission at the 4-month follow-up. Phenomenal improvement was observed in as little as 3 months after treatment, as shown in Figure 1 and Figure 2. Unlike the case described by K.S. Matthews et al., conservative management of the cervical necrosis did not fail, and has led to excellent results. This complication should not be evaluated by laparoscopic examination, as suggested by Marnitz et al., unless conservative treatment has been tried. This exploration would have added an unnecessary risk of complications, as well as surgical debridement as proposed by Güth et al. Thus, a conservative management is a safe and effective treatment for cervical necrosis following radiation therapy, and such management should be considered rather than a laparoscopic examination and surgical intervention which are more invasive and could result in chronic disability. Hyperbaric oxygen therapy has been used for cervical cancer patients with delayed radiation therapy damages, but was not necessary in the patients described in this study [18].

Given the retrospective nature of our study, a recall bias may exist. Furthermore, some patients could have gone to an outpatient clinic or to the emergency to manage the cervical necrosis without being seen by the Radiation Oncology Department, which might limit the generalizability of our results for the percentage of CN after RT. However, this study was conducted in a University Hospital Center with a policy to send the patients to the belonging department, and there is a close working relationship between the radiation oncologists and the gynecologic oncologists at the center. This case series was also performed at a single care center, which provides a consistency in the care and the management of the necrosis for the patients.

## Conclusions

In conclusion, clinicians should be aware that a conservative management for the treatment of cervical necrosis in patients receiving radiation therapy for cervical cancer is effective, and should be attempted. This complication is more common in smokers, and cessation of tobacco smoking should result in fewer complications of CN.

## Consent

Informed consent was obtained from the patients for the publication of this report and accompanying images.

## Competing interests

The authors declared that they have no competing interests.

## Authors’ contributions

ZSF collected and analyzed the data, and wrote the manuscript. TVN and MB treated the vast majority of the patients referenced in the study, participated in the coordination and supervision of the study and helped review the manuscript. All authors read and approved the final manuscript.
